# Patterns of asthma medication use and its association with periodontitis: A nationwide population-based study

**DOI:** 10.1097/MD.0000000000049852

**Published:** 2026-07-24

**Authors:** Su-Na Lee, Ki-Ho Chung

**Affiliations:** a Department of Preventive and Public Health Dentistry, Chonnam National University School of Dentistry, Gwangju, Republic of Korea; b Dental Science Research Institute, Chonnam National University, Gwangju, Republic of Korea.

**Keywords:** asthma, health behavior, oral hygiene, periodontitis, steroids

## Abstract

With the increasing prevalence of asthma, the association between periodontitis and the increasing use of inhalers deserves attention. This study determines the association between asthma medication administration and periodontitis among Korean adults. In total, 1185 out of 72,751 individuals with asthma from the 2010 to 2018 National Health and Nutrition Examination Survey, a large healthcare dataset, were analyzed. Frequency analysis and chi-square tests were performed to identify the gender-based status of asthma medication in patients with asthma. The association of asthma medication status with periodontitis was evaluated using multivariable logistic regression analysis, and the gender-based correlation was confirmed through subgroup divisions. Taking asthma medication as needed exhibited an odds ratio (OR) > 1.5 in the overall population (OR = 1.54; 95% confidence interval: 1.03–2.30), and the OR for periodontitis risk among men with asthma was >2. Moreover, in the model controlling for all confounding factors, male patients with asthma who took asthma medication “when necessary” had a 2.10-fold (95% confidence interval = 1.06–4.19) increased prevalence of periodontitis compared to “No” medication controls. This study found a significant association between as-needed asthma medication use and a higher prevalence of periodontitis, particularly among men with asthma.

## 1. Introduction

Asthma is a chronic inflammatory disease of the respiratory tract characterized by chronic inflammation, hypersensitivity, and intermittent airway constriction.^[1,2]^ Asthma can cause breathing difficulties, wheezing, coughing, and tightness in the chest, leading to difficulties in daily activities and even fatalities in cases of severe asthma attacks.^[3,4]^ The rising incidence of asthma has been associated with environmental factors, such as the increasing prevalence of allergic diseases and worsening air pollution.^[5]^ Previous studies in Korea have reported that asthma healthcare costs are steadily increasing, with a higher prevalence observed among females than males.^[6]^ Additionally, asthma mortality rates have increased from 16.2% to 28.0% per 1,00,000 individuals, with a higher proportion observed in males.^[6]^ Approximately 4,00,000 individuals died of asthma between 1990 and 2015 globally.^[7]^ Periodontitis is a chronic inflammatory disease characterized by the destruction of periodontal tissues and subsequent tooth loss due to the formation of biofilm (dental plaque) on the surface and host responses.^[8]^ These diseases are associated with various systemic conditions, with their effects mediated by periodontitis-causing bacteria and their toxins circulating in the bloodstream, as well as by inflammatory mediators produced at periodontal sites that travel through the bloodstream to affect various tissues.^[9]^ The main factor responsible for reducing the occurrence of periodontitis is the interaction between bacterial and immunological factors, of which saliva is an important factor influencing the severity of periodontitis.^[10]^ However, medications, such as inhaled corticosteroids (ICS), used to treat or control asthma can affect salivary secretion, which can adversely affect periodontal health.^[11]^ Various medications have been developed and used in clinical practice to treat asthma. In Korea, oral medications were preferred over inhalers until a few years ago; however, the use of inhalers has been increasing since the Global Initiatives for Asthma guidelines were revised in 2019 to prioritize ICS as a rescue treatment for all stages of asthma.^[12]^ Altered salivary composition, decreased salivary volume due to inhalant use, and mouth breathing in asthmatics are strong risk factors for periodontitis.^[13,14]^

Although studies have been conducted on asthma and periodontitis,^[15]^ further studies are required to investigate the relationship between medication usage patterns and prevalence rates. Large-scale studies on the risk of periodontitis are warranted because of the lack of a large representative sample study, considering the slow onset nature of periodontitis. Therefore, in a nationwide population-based sample, this study aimed to evaluate the association between asthma medication use behavior and periodontitis in adults, adjusted for various covariates.

## 2. Materials and methods

### 2.1. Korea National Health and Nutrition Examination Survey (KNHANES)

KNHANES is a nationwide survey that has been monitoring the health and nutritional status of Koreans since 1998. Conducted by the Korea Centers for Disease Control and Prevention under the National Health Promotion Act, it gathers data from around 10,000 individuals annually. The survey collects information on factors such as socioeconomic status, health behaviors, quality of life, healthcare use, and noncommunicable disease profiles through 3 components: health interviews, health examinations, and nutrition surveys. Health interviews and examinations are conducted by trained professionals at mobile centers, while dieticians visit participants’ homes for follow-ups.^[16]^

### 2.2. Research participants

**Figure 1.**
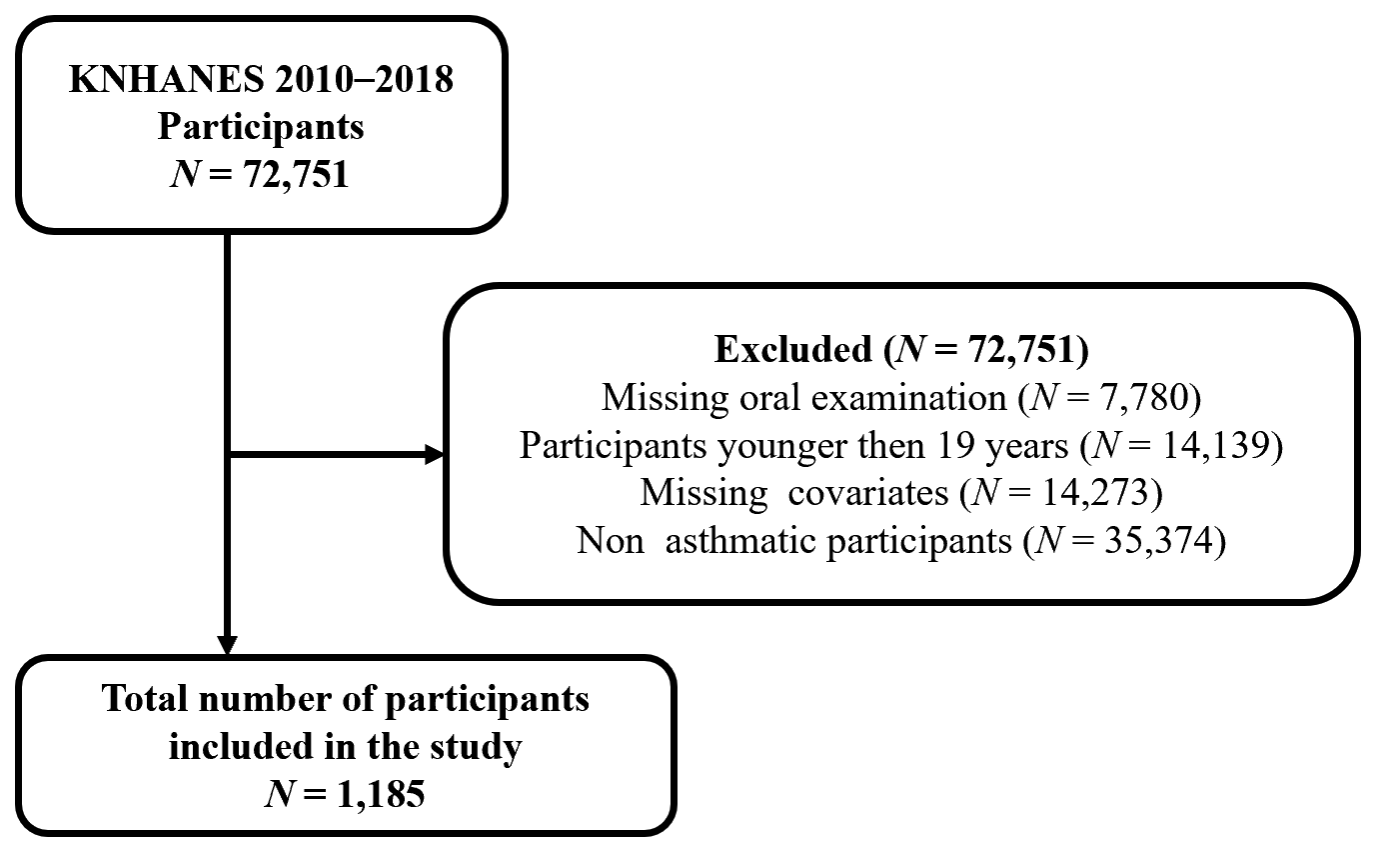
Flow chart of the selection process for the study population.

Data were obtained from the 5th (2010–2013), 6th (2014–2015), and 7th (2016–2018) KNHANES. Of the 92,606 eligible patients for screening and health surveys, 72,751 responded, yielding a response rate of 78.6%. Of these, 1185 people with asthma were included in the study after excluding those with missing values for sociodemographic characteristics, general health status characteristics, oral health behavior characteristics, oral examination variables, and asthma medication status (Fig. 1). Participants were categorized based on asthma treatment pattern (regular, when necessary, no treatment), and their demographic and oral health characteristics were evaluated (see Tables S1 and S2, Supplemental Digital Content 1 for complete distributions). Baseline demographic and oral health characteristics of non-asthmatic participants were compared to provide control group context; detailed distributions are reported in Tables S3 and S4, Supplemental Digital Content 2.

KNHANES was approved by the Centers for Disease Control and Prevention Institutional Review Board (2010-02CON-21-C through 2018-01-03-P-A), and written informed consent was obtained from all participants prior to the study.

## 3. Study variables

### 3.1. Asthma definition and treatment categories

Participants were classified as having asthma if they reported a physician diagnosis of asthma in the health interview. Asthma pharmacotherapy during the past 12 months was categorized using the KNHANES medication-use item as follows: “Regularly” if the participant reported regular treatment to prevent exacerbations and control symptoms; “When necessary” if the participant reported treatment only when symptoms occurred; and “No medication” if the participant had a physician diagnosis of asthma but reported no use of any prescribed asthma medication in the past 12 months. Responses of “don’t know/refused/missing” were excluded from the analytic sample. These operational definitions were prespecified prior to analysis.

### 3.2. Periodontitis

KNHANES follows a standardized method for conducting oral examinations on participants by trained dentists. Periodontitis was assessed by a dentist using the Community Periodontal Index (CPI) according to WHO (1982) guidelines.^[17]^ CPI scores were categorized as follows: CPI 0 = normal healthy periodontal tissue; CPI 1 = periodontal bleeding on probing; CPI 2 = the presence of calculus at the gingival margin or subgingival; CPI 3 = pathologic periodontal pockets with a depth of 3.5 to 5 mm; CPI 4 = pathologic periodontal pockets with a depth of 5.5 mm or more. A CPI score of 3 or higher was defined as the presence of periodontitis. This aligns with a previous study.^[18]^

### 3.3. General and socioeconomic characteristics

This study used modified and supplemented covariates used in previous studies.^[18]^ Age, income level, and educational level were selected as demographic and socioeconomic characteristic variables. Ages were classified in 10-year increments. Income level was divided into upper, upper-middle, middle-lower, and lower based on household income, whereas educational level was categorized as elementary school graduate or below, middle school graduate, high school graduate, and university graduate or above.

Variables related to general health behaviors included smoking and alcohol consumption. Smoking status was defined based on lifetime smoking experience as having smoked less than 5 packs (100 cigarettes) and having smoked 5 packs or more (100 cigarettes), and no smoking experience for those who had never smoked. Alcohol consumption was categorized as follows: never in the past year, less than once a month (nondrinker), about once a month, 2 to 4 times a month (low drinker), 2 to 3 times a week (moderate drinker), and >4 times a week (heavy drinker).

### 3.4. Oral health characteristics

Oral health behavior variables included oral examination in the past year, daily brushing, use of oral care products, self-perceived oral health status, problems while chewing, and speech problems. The presence of oral examinations was categorized as yes or no; daily brushing was categorized as ≥2 times/d or ≤1 time/d; and oral care products were categorized as “yes” if at least one of the following was used: interdental toothbrush and/or floss. Self-perceived oral health was categorized as “good” for very good, good, and fair, and “poor” for poor and very poor. Chewing and speech problems are categorized as “Yes” for very uncomfortable and uncomfortable and “No” for tolerable, not uncomfortable, and not at all uncomfortable.

## 4. Analysis methods

Due to the complex sampling design of KNHANES, a complex sample data analysis that considered strata, clusters, and weights was conducted. First, frequency analysis and chi-square tests were performed to determine the gender-dependent differences in demographic characteristics and general health behaviors. Next, frequency analysis and chi-square tests were conducted to determine the differences in oral health behavior of the participants by gender. Additionally, these tests were performed to determine the asthma medication status of patients with asthma by gender. To determine the effect of asthma medication status on periodontitis (dichotomization) in the patients, multivariable logistic regression analysis was performed by constructing 4 models. The statistical test set the significance level at *P* < .05. Statistical analyses were performed using SAS 9.4 software (SAS Institute, Cary).

## 5. Results

### 5.1. General/socioeconomic characteristics

Of the 1185 patients with asthma, 43.55% were male and 56.45% were female, indicating a slightly higher proportion of female patients. The mean age was 43.28 and 51.63 years for male and female patients, with females being significantly older (*P* < .05). There was statistical significance between males and females in terms of education level and household income, smoking, and alcohol consumption (*P* < .05, Table 1).

**Table 1:** General characteristics of asthma-diagnosed group.

Variables	Category	Male asthma-diagnosed group (n = 453)		Female asthma-diagnosed group (n = 732)		Total asthma-diagnosed group (n = 1185)		*P*-value
		N	%	N	%	N	%	
Age	19–29	98	21.63	177	24.18	275	23.21	<.001*
	30–39	70	15.45	165	22.54	235	19.83	
	40–49	49	10.82	130	17.76	179	15.11	
	50–59	52	11.48	174	23.77	226	19.07	
	60–69	86	18.98	253	34.56	339	28.61	
	≥70	98	21.63	286	39.07	384	32.41	
Education	≤Elementary school	110	24.28	228	31.15	338	28.52	.0207*
	Middle school	117	25.83	158	21.58	275	23.21	
	High school	108	23.84	173	23.63	281	23.71	
	≥University or College	118	26.05	173	23.63	291	24.56	
Household income	Low	110	24.28	315	43.03	425	35.86	<.001*
	Middle-low	54	11.92	79	10.79	133	11.22	
	Middle-high	142	31.35	165	22.54	307	25.91	
	High	147	32.45	173	23.63	320	27.00	
Smoking	Never smoker	110	24.28	627	85.66	737	62.19	<.001*
	Current smoker (former smoker)	343	75.72	105	14.34	448	37.81	
Alcohol consumption	Nondrinker	145	32.01	494	67.49	639	53.92	<.001*
	1/mo	153	33.77	177	24.18	330	27.85	
	≥2/mo	155	34.22	61	8.33	216	18.23	

* Statistically significant.

### 5.2. Oral health care characteristics of asthma patients

In terms of oral health behaviors, there was a significant difference between male and female patients with asthma in brushing frequency and number of remaining teeth (*P* < .05), whereas the rest of the variables did not show significant differences (*P* > .05, Table 2).

**Table 2:** Oral health characteristics of asthma-diagnosed group.

Variables	Category	Male asthma-diagnosed group (n = 453)		Female asthma-diagnosed group (n = 732)		Total asthma-diagnosed group (n = 1185)		*P*-value
		N	%	N	%	N	%	
Teeth brushing	<2	87	19.21	88	12.02	175	14.77	.0234*
	≥2	366	80.79	644	87.98	1010	85.23	
Dental checkup	Yes	118	26.05	200	27.32	318	26.84	.2253
	No	335	73.95	532	72.68	867	73.16	
Chewing difficulty	Yes	122	26.93	236	32.24	358	30.21	.16
	No	331	73.07	496	67.76	827	69.79	
Speaking difficulty	Yes	52	11.48	105	14.34	157	13.25	.0677
	No	401	88.52	627	85.66	1028	86.75	
Self-perception	Good	234	51.66	369	50.41	603	50.89	.7071
	Bad	219	48.34	363	49.59	582	49.11	
Periodontitis	Yes	142	31.35	194	26.50	336	28.35	.4439
	No	311	68.65	538	73.50	849	71.65	
Present tooth	<20	85	18.76	158	21.58	243	20.51	.0239*
	≥20	368	81.24	574	78.42	942	79.49	

* Statistically significant.

### 5.3. Asthma medication use by male and female patients

There was no significant difference between male and female patients with asthma in terms of asthma medication use (*P* > .05, Table 3).

**Table 3:** Medication frequency of asthma-diagnosed group.

Variables	Category	Male asthma-diagnosed group (n = 453)			Female asthma-diagnosed group (n = 732)			Total asthma-diagnosed group (n = 1185)			*P*-value
		N	%		N	%		N	%		
Medication frequency	No	266	58.72	359		49.044	625		52.74	.0659	
	When necessary	107	23.62	240		32.787	347		29.28		
	Regularly	80	17.66	133		18.169	213		17.97		

### 5.4. Association between asthma medication usage status and oral health

The prevalence of periodontitis according to asthma medication use was evaluated using logistic regression models (Table 4). In the overall population, participants who used asthma medication “when necessary” showed increased odds of periodontitis across all models (odds ratios ranging from 1.53 to 2.00), and these associations remained statistically significant after adjustment, as all 95% confidence intervals excluded 1.0. Among male patients with asthma, the “when necessary” medication group consistently exhibited more than a more than 2-fold increase in the odds of periodontitis across all models (odds ratios ranging from 2.10 to 2.89), with statistically significant associations observed in every model. In the fully adjusted model, male patients who used asthma medication “when necessary” had a 2.10-fold higher prevalence of periodontitis (95% confidence interval: 1.06–4.19) compared with those who did not use asthma medication. After adjustment, regular asthma medication use was not significantly associated with periodontitis in either sex. Sensitivity analysis using ≥2 times/d as the cutoff yielded similar association patterns to those obtained using the original ≥3 times/d definition (Table S5, Supplemental Digital Content 3), supporting the robustness of the findings.

**Table 4:** Association between antiasthmatic treatment and periodontitis.

Antiasthmatic medication		Model 1 OR (95% CI)	Model 2 OR (95% CI)	Model 3 OR (95% CI)	Model 4 OR (95% CI)
Total	No	1	1	1	1
	When necessary	2.00 (1.38–2.91)	1.65 (1.1–2.48)	1.53 (1.03–2.27)	1.54 (1.03–2.30)
	Regularly	1.81 (1.14–2.87)	1.05 (0.63–1.74)	1.07 (0.64–1.78)	1.03 (0.62–1.70)
Male	No	1	1	1	1
	When necessary	2.89 (1.56–5.34)	2.48 (1.29–4.77)	2.35 (1.23–4.5)	2.10 (1.06–4.19)
	Regularly	1.83 (0.84–4.01)	0.92 (0.37–2.33)	0.91 (0.38–2.21)	0.75 (0.34–1.63)
Female	No	1	1	1	1
	When necessary	1.57 (0.98–2.52)	1.23 (0.75–2.03)	1.12 (0.69–1.82)	1.11 (0.68–1.82)
	Regularly	1.79 (1.03–3.13)	1.11 (0.63–1.96)	1.08 (0.62–1.89)	1.08 (0.62–1.87)

Results represent ORs for periodontitis (95% confidence interval [CI]). Model 1: not adjusted; model 2: adjusted for sex, age; model 3: adjusted for sex, age, family income, education, smoking, alcohol variables; model 4: adjusted for sex, age, family income, education, smoking, alcohol, tooth brushing, dental checkup, chewing difficulty, speaking difficulty, self-perception, and present tooth variables.

CI = confidence interval, OR = odds ratio.

## 6. Discussion

The study used data representative of the Korean population to investigate the association between medication use among patients with asthma and periodontitis. Previous studies have reported a positive association between asthma and oral health problems, indicating that individuals with asthma are more likely to experience periodontal disease and compromised oral health status.^[19,20]^ The study concluded that the risk of periodontitis was higher in patients who take their asthma medication only when necessary. There is a correlation between periodontitis and respiratory diseases, and maintaining healthy gums can improve lung function and reduce the frequency of respiratory attacks caused by asthma.^[21,22]^ Our study is significant given the lack of large-scale cross-sectional studies of asthma medication usage patterns,^[18,21]^ although there have been studies that have demonstrated a relationship between asthma and periodontitis.

The results of this study indicated that male patients with asthma accounted for 43.55%, whereas female patients with asthma accounted for 56.45%, supporting the results reported by Lee et al^[6]^ and Yun et al,^[5]^ indicating a higher prevalence of asthma in adult females. The proportion of nonsmokers was 85.66% in female patients and 24.28% in male patients, with a lower proportion of smoking in females, consistent with previous studies by Yoon et al^[15]^ and Lee et al,^[23]^ suggesting a weak association between asthma and smoking. The prevalence of asthma according to alcohol consumption was 67.49% in women who did not consume any alcohol, and 32.01% in men, a significant difference, but the prevalence of asthma according to alcohol consumption varied between studies, suggesting that further research is needed.^[20,24]^ In terms of general behavior, asthma prevalence was higher among females, despite lower levels of income and education, whereas rates of smoking and alcohol consumption, which negatively impact overall health, were lower among them.

In the study, the oral healthcare characteristics of patients with asthma showed that 46.99% of female brushed at least thrice a day compared to 38.19% of male, and 78.42% of female had 20 or fewer remaining teeth compared to 81.24% of male, indicating that male patients with asthma had poorer oral health. This study also observed lower levels of oral hygiene management among male patients with asthma compared to findings by Rubbaey,^[25]^ who reported higher rates of dental caries and poorer oral cleanliness among patients with asthma, particularly men. Similarly, Hong^[26]^ reported higher smoking rates and poorer oral hygiene among males, suggesting that despite higher asthma prevalence among females, factors influencing the association with periodontitis are more pronounced among male patients.

To determine the impact of asthma medication usage patterns on periodontitis in this study, multivariable logistic regression analysis was conducted, and the odds ratio for “asthma medication only as needed” was >1.5 in all models for the entire population. Asthma medications are divided into disease modifiers, which are used on a daily basis, and symptom relievers, which are used in emergencies. Symptom relievers have a short duration of action and are fast-acting, and combination medications containing ICS + long-acting β2-agonists are recommended.^[27]^ The results of this study were consistent with the results of Shen,^[28]^ who reported a higher risk of periodontitis among patients with poor asthma control and frequent healthcare utilization. However, because information on specific medication classes was not available in the present study, the observed association should not be interpreted as evidence of an effect of any particular asthma medication. Kim^[29]^ reported that patients with systemic diseases and poor medication adherence were more likely to utilize emergency services. Therefore, the association observed in the “when necessary” medication group may reflect differences in disease management behaviors or asthma control status rather than the effects of a specific medication class. This is consistent with the studies by Vittorio et al^[15]^ and Yaghobee et al,^[30]^ who reported that taking asthma medications negatively affects periodontal health.

The results of this study suggest that the asthma medication only “when necessary” behavior and the periodontitis crossover ratio were only significant in men and women, which could be explained by lower adherence among those who take medication “when necessary.” Adherence is a broad concept that encompasses medication adherence in chronic diseases. Individuals reporting medication use only when necessary may represent a subgroup with different treatment behaviors or asthma-management patterns compared with those using medication more regularly. In addition, the results of Kim^[31]^ comparing medication adherence between males and females showed that men exhibited lower medical adherence than women. Second, it is suggested that the association is related to the oral health management behavior of men compared with women. Men are less likely to brush their teeth thrice a day and have fewer remaining teeth, indicating a neglect of oral health management. It is also possible that factors related to inhaler use, such as changes in salivary flow, oral moisture, or oral hygiene practices, may contribute to periodontal health; however, these mechanisms could not be directly evaluated in the present study.

Long-acting β2-agonists are recommended for use in combination with ICSs because they can improve symptoms in >90% of asthma patients and reduce the need for into short-acting β2-agonists^[32,33]^. ICSs are known to have local side effects, such as dysphonia, oral candidiasis, and dry mouth.^[34,35]^ This is more common at higher daily doses.^[36,37]^

Studies by Srensson et al^[38]^ and Shashikiran^[39]^ have reported that asthma medications affect periodontitis and suggested that asthma inhalers may increase plaque accumulation and calculus by reducing oral pH and saliva production.^[40,41]^ Poor oral care in combination with asthma medication is associated with a higher prevalence of periodontitis and other oral diseases.

Kang et al^[42]^ reported reported that patients with asthma frequently experience coughing and mouth breathing, both of which may influence the oral environment. Cecchin-Albertoni et al^[43]^ reported that reduced salivary flow compromises oral functions such as mastication, swallowing, and speech, which may lead to digestive disorders and increased susceptibility to gingivitis and caries.

Given the higher prevalence of periodontitis observed among participants who reported using asthma medication only when necessary, there is a need for dental professionals to actively support oral health care and education in patients with asthma, particularly males. Because medication class and inhaler type were not available in the present dataset, these findings should not be interpreted as evidence of an adverse effect of any specific asthma medication.

The strength of this study lies in its use of nationally representative KNHANES healthcare big data to assess the risk of periodontitis among patients with asthma. However, there are several limitations to this study that should be considered. First, the National Health and Nutrition Examination Survey is an instance-based data set, making it difficult to make clear causal inferences. Second, due to the absence of detailed information on medication class, dose, and duration in KNHANES, ICS-specific analyses could not be conducted. As inhaler-use frequency was used as a proxy measure of controller use, the findings should be interpreted with caution. Future prospective studies with clinician-verified prescription data and standardized oral-health parameters are warranted to elucidate dose–response associations and temporal causality. Third, this study is a cross-sectional study, and a follow-up study with long-term follow-up is warranted.

The results of this study indicate that asthma medication use only when necessary, particularly among males, was associated with a higher prevalence of periodontitis. These findings highlight the importance of oral health promotion and periodontal monitoring among patients with asthma and suggest that oral health education may be particularly beneficial for those reporting medication use only when necessary.

## Author contributions

**Conceptualization:** Ki-Ho Chung.

**Data curation:** Su-Na Lee, Ki-Ho Chung.

**Formal analysis:** Su-Na Lee, Ki-Ho Chung.

**Funding acquisition:** Ki-Ho Chung.

**Investigation:** Su-Na Lee.

**Methodology:** Su-Na Lee, Ki-Ho Chung.

**Supervision:** Ki-Ho Chung.

**Validation:** Su-Na Lee, Ki-Ho Chung.

**Visualization:** Su-Na Lee, Ki-Ho Chung.

**Writing – original draft:** Su-Na Lee, Ki-Ho Chung.

**Writing – review & editing:** Ki-Ho Chung.










