# Supplementary material for: Patterns of asthma medication use and its association with periodontitis: A nationwide population-based study
Source: Medicine (Baltimore). 2026 Jul 24;105(30):e49852. doi: 10.1097/MD.0000000000049852 (PMC13406317; doi:10.1097/MD.0000000000049852)
Supplement: Supplementary file 1 [file medi-105-e49852-s001.docx]

Supplementary Table 1. Baseline characteristics of asthma participants according to treatment pattern

| Variables | Category | Regular treatment (n = 213) | | When necessary treatment. (n = 347) | | No treatmen (n = 625) | | P-value |
| --- | --- | --- | --- | --- | --- | --- | --- | --- |
|  |  | N | % | N | % | N | % |  |
| Age | 19–29 | 4 | 1.88 | 34 | 9.80 | 139 | 22.24 | <.0001* |
|  | 30–39 | 14 | 6.57 | 45 | 12.97 | 106 | 16.96 |  |
|  | 40–49 | 21 | 9.86 | 42 | 12.10 | 67 | 10.72 |  |
|  | 50–59 | 32 | 15.02 | 60 | 17.29 | 82 | 13.12 |  |
|  | 60–69 | 53 | 24.88 | 83 | 23.92 | 117 | 18.72 |  |
|  | ≥70 | 89 | 41.78 | 83 | 23.92 | 114 | 18.24 |  |
| Education | ≤Elementary school | 108 | 50.70 | 138 | 39.77 | 179 | 28.64 | 0.0004* |
|  | Middle school | 26 | 12.21 | 43 | 12.39 | 64 | 10.24 |  |
|  | High school | 38 | 17.84 | 89 | 25.65 | 180 | 28.80 |  |
|  | ≥University or College | 41 | 19.25 | 77 | 22.19 | 202 | 32.32 |  |
| Household Income | Low | 91 | 42.72 | 111 | 31.99 | 136 | 21.76 | <.0001* |
|  | Middle-Low | 51 | 23.94 | 83 | 23.92 | 141 | 22.56 |  |
|  | Middle-High | 39 | 18.31 | 75 | 21.61 | 167 | 26.72 |  |
|  | High | 32 | 15.02 | 78 | 22.48 | 181 | 28.96 |  |
| Smoking | Never smoker | 131 | 61.50 | 225 | 64.84 | 381 | 60.96 | 0.9005 |
|  | Current smoker (Former smoker) | 82 | 38.50 | 122 | 35.16 | 244 | 39.04 |  |
| Alcohol Consumption | Nondrinker | 143 | 67.14 | 194 | 55.91 | 302 | 48.32 | 0.0002* |
|  | 1 per month | 40 | 18.78 | 85 | 24.50 | 205 | 32.80 |  |
|  | ≥2 per month | 30 | 14.08 | 68 | 19.60 | 118 | 18.88 |  |

* Statistically significant
