# Supplementary material for: Patterns of asthma medication use and its association with periodontitis: A nationwide population-based study
Source: Medicine (Baltimore). 2026 Jul 24;105(30):e49852. doi: 10.1097/MD.0000000000049852 (PMC13406317; doi:10.1097/MD.0000000000049852)
Supplement: Supplementary file 2 [file medi-105-e49852-s002.docx]

Supplementary Table 3. Baseline characteristics of people without asthma

| Variables | Category | Male (n = 15114) | | Female (n = 20206) | | Total (n = 35320) | | P-value |
| --- | --- | --- | --- | --- | --- | --- | --- | --- |
|  |  | N | % | N | % | N | % |  |
| Age | 19–29 | 1968 | 13.02 | 2465 | 12.20 | 4433 | 12.55 | <.0001* |
|  | 30–39 | 2608 | 17.26 | 3616 | 17.90 | 6224 | 17.62 |  |
|  | 40–49 | 2873 | 19.01 | 3847 | 19.04 | 6720 | 19.03 |  |
|  | 50–59 | 2899 | 19.18 | 4117 | 20.38 | 7016 | 19.86 |  |
|  | 60–69 | 2670 | 17.67 | 3306 | 16.36 | 5976 | 16.92 |  |
|  | ≥70 | 2096 | 13.87 | 2855 | 14.13 | 4951 | 14.02 |  |
| Education | ≤Elementary school | 2194 | 14.52 | 5243 | 25.95 | 7437 | 21.06 | <.0001* |
|  | Middle school | 1618 | 10.71 | 2037 | 10.08 | 3655 | 10.35 |  |
|  | High school | 5464 | 36.15 | 6576 | 32.54 | 12040 | 34.09 |  |
|  | ≥University or College | 5838 | 38.63 | 6350 | 31.43 | 12188 | 34.51 |  |
| Household Income | Low | 2425 | 16.04 | 3819 | 18.90 | 6244 | 17.68 | <.0001* |
|  | Middle-Low | 3737 | 24.73 | 5163 | 25.55 | 8900 | 25.20 |  |
|  | Middle-High | 4358 | 28.83 | 5538 | 27.41 | 9896 | 28.02 |  |
|  | High | 4594 | 30.40 | 5686 | 28.14 | 10280 | 29.11 |  |
| Smoking | Never smoker | 3415 | 22.59 | 18088 | 89.52 | 21503 | 60.88 | <.0001* |
|  | Current smoker (Former smoker) | 11699 | 77.41 | 2118 | 10.48 | 13817 | 39.12 |  |
| Alcohol Consumption | Nondrinker | 4172 | 27.60 | 12141 | 60.09 | 16313 | 46.19 | <.0001* |
|  | 1 per month | 5464 | 36.15 | 5994 | 29.66 | 11458 | 32.44 |  |
|  | ≥2 per month | 5478 | 36.24 | 2071 | 10.25 | 7549 | 21.37 |  |

* Statistically significant
