# Supplementary material for: Patterns of asthma medication use and its association with periodontitis: A nationwide population-based study
Source: Medicine (Baltimore). 2026 Jul 24;105(30):e49852. doi: 10.1097/MD.0000000000049852 (PMC13406317; doi:10.1097/MD.0000000000049852)
Supplement: Supplementary file 3 [file medi-105-e49852-s003.docx]

Supplementary Table 5. Association between antiasthmatic treatment and periodontitis according to tooth brushing frequency (≥3 times/day vs ≤3 times/day)

| Antiasthmatic medication | | Model 1  OR (95% CI) | Model 2  OR (95% CI) | Model 3  OR (95% CI) | Model 4  OR (95% CI) |
| --- | --- | --- | --- | --- | --- |
| Total | No | 1 | 1 | 1 | 1 |
|  | When necessary | 2.00 (1.38–2.91) | 1.65 (1.1–2.48) | 1.53 (1.03–2.27) | 1.5 (1.01–2.25) |
|  | Regularly | 1.81 (1.14–2.87) | 1.05 (0.63–1.74) | 1.07 (0.64–1.78) | 1.01 (0.61–1.68) |
| Male | No | 1 | 1 | 1 | 1 |
|  | When necessary | 2.89 (1.56–5.34) | 2.48 (1.29–4.77) | 2.35 (1.23–4.5) | 2.09 (1.05–4.18) |
|  | Regularly | 1.83 (0.84–4.01) | 0.92 (0.37–2.33) | 0.91 (0.38–2.21) | 0.75 (0.35–1.63) |
| Female | No | 1 | 1 | 1 | 1 |
|  | When necessary | 1.57 (0.98–2.52) | 1.23 (0.75–2.03) | 1.12 (0.69–1.82) | 1.09 (0.67–1.78) |
|  | Regularly | 1.79 (1.03–3.13) | 1.11 (0.63–1.96) | 1.08 (0.62–1.89) | 1.04 (0.6–1.81) |

* Results represent ORs for periodontitis [95% confidence interval (CI)]. Model 1: not adjusted; model 2: adjusted for sex, age; model 3: adjusted for sex, age, family income, education, smoking, alcohol variables; model 4: adjusted for sex, age, family income, education, smoking, alcohol, tooth brushing, dental check-up, chewing difficulty, speaking difficulty, self-perception and present tooth variables. OR, odds ratio
