# Supplementary material for: Patterns of asthma medication use and its association with periodontitis: A nationwide population-based study
Source: Medicine (Baltimore). 2026 Jul 24;105(30):e49852. doi: 10.1097/MD.0000000000049852 (PMC13406317; doi:10.1097/MD.0000000000049852)
Supplement: Supplementary file 4 [file medi-105-e49852-s004.docx]

Supplementary Table 2. Oral health characteristics of asthma participants according to treatment pattern

| Variables | Category | Regular treatment (n = 213) | | When necessary treatment. (n = 347) | | No treatmen (n = 625) | | P-value |
| --- | --- | --- | --- | --- | --- | --- | --- | --- |
|  |  | N | % | N | % | N | % |  |
| Teeth brushing | <2 | 38 | 17.84 | 58 | 16.71 | 79 | 12.64 | 0.1995 |
|  | ≥2 | 175 | 82.16 | 289 | 83.29 | 546 | 87.36 |  |
| Dental check-up | Yes | 58 | 27.23 | 95 | 27.38 | 165 | 26.40 | 0.6210 |
|  | No | 155 | 72.77 | 252 | 72.62 | 460 | 73.60 |  |
| Chewing difficulty | Yes | 84 | 39.44 | 119 | 34.29 | 155 | 24.80 | 0.0011* |
|  | No | 129 | 60.56 | 228 | 65.71 | 470 | 75.20 |  |
| Speaking difficulty | Yes | 42 | 19.72 | 55 | 15.85 | 60 | 9.60 | 0.0346* |
|  | No | 171 | 80.28 | 292 | 84.15 | 565 | 90.40 |  |
| Self-perception | Good | 97 | 45.54 | 169 | 48.70 | 337 | 53.92 | 0.2902 |
|  | Bad | 116 | 54.46 | 178 | 51.30 | 288 | 46.08 |  |
| Periodontitis | Yes | 73 | 34.27 | 111 | 31.99 | 152 | 24.32 | 0.0004* |
|  | No | 140 | 65.73 | 236 | 68.01 | 473 | 75.68 |  |
| Present tooth | <20 | 60 | 28.17 | 79 | 22.77 | 104 | 16.64 | 0.0217 |
|  | ≥20 | 153 | 71.83 | 268 | 77.23 | 521 | 83.36 |  |

* Statistically significant
