# Supplementary material for: Patterns of asthma medication use and its association with periodontitis: A nationwide population-based study
Source: Medicine (Baltimore). 2026 Jul 24;105(30):e49852. doi: 10.1097/MD.0000000000049852 (PMC13406317; doi:10.1097/MD.0000000000049852)
Supplement: Supplementary file 5 [file medi-105-e49852-s005.docx]

Supplementary Table 4. Oral health characteristics of people without asthma

| Variables | Category | Male (n = 15114) | | Female (n =20206 ) | | Total (n = 35320) | | P-value |
| --- | --- | --- | --- | --- | --- | --- | --- | --- |
|  |  | N | % | N | % | N | % |  |
| Teeth brushing | <2 | 2360 | 15.61 | 1476 | 7.30 | 3836 | 10.86 | <.0001* |
|  | ≥2 | 12754 | 84.39 | 18730 | 92.70 | 31484 | 89.14 |  |
| Dental check-up | Yes | 4655 | 30.80 | 6061 | 30.00 | 10716 | 30.34 | 0.7527 |
|  | No | 10459 | 69.20 | 14145 | 70.00 | 24604 | 69.66 |  |
| Chewing difficulty | Yes | 3549 | 23.48 | 4580 | 22.67 | 8129 | 23.02 | 0.7806 |
|  | No | 11565 | 76.52 | 15626 | 77.33 | 27191 | 76.98 |  |
| Speaking difficulty | Yes | 1237 | 8.18 | 1649 | 8.16 | 2886 | 8.17 | 0.3914 |
|  | No | 13877 | 91.82 | 18557 | 91.84 | 32434 | 91.83 |  |
| Self-perception | Good | 8393 | 55.53 | 11774 | 58.27 | 20167 | 57.10 | <.0001* |
|  | Bad | 6721 | 44.47 | 8432 | 41.73 | 15153 | 42.90 |  |
| Periodontitis | Yes | 5579 | 36.91 | 4977 | 24.63 | 10556 | 29.89 | <.0001* |
|  | No | 9535 | 63.09 | 15229 | 75.37 | 24764 | 70.11 |  |
| Present tooth | <20 | 2065 | 13.66 | 2505 | 12.40 | 4570 | 12.94 | 0.3761 |
|  | ≥20 | 13049 | 86.34 | 17701 | 87.60 | 30750 | 87.06 |  |

* Statistically significant
